# One Small Step Towards Fixing a Broken System

**DOI:** 10.5334/cpsy.148

**Published:** 2025-05-13

**Authors:** Xiaosi Gu, Rick A. Adams

**Affiliations:** 1 Department of Psychiatry & Department of Neuroscience, Icahn School of Medicine at Mount Sinai, New York, NY, US; 2 Department of Psychiatry & Department of Biomedical Informatics and Data Science, Yale School of Medicine, New Haven, CT, US; 3 Institute of Cognitive Neuroscience and Department of Computer Science, University College London, London, UK

Peer review is a cornerstone of the scientific publishing ecosystem. The growth of modern science has heavily relied on the contributions of many, many generous reviewers whose critiques have provided important independent opinions and improved the quality of the scientific study being reviewed. However, with the exponential growth of research and the fast-changing economic landscape of scientific publishing, the current model of peer review is becoming increasingly unsustainable. Reviewers are often overloaded with requests from journals and editors often struggle to secure high quality reviews in a timely manner. In a recent submission one of us handled for a different journal, 10 out of 12 invited reviewers turned down the invitation. Many factors might have contributed to the lack of motivation for reviewers to accept review invitations – too busy, a conflict of interest, etc. However, when rejections happen at a larger scale, we might want to systematically examine some of the root causes for the peer review system that seems to be on the brink of a breakdown – what can we do to fix the system?

When analyzing the peer review system with a standard economic framework, one obvious issue that jumps out right away is the following – why are reviewers not compensated for their time, as is the case for any other type of labor market? Traditionally, it is considered a researcher’s responsibility to volunteer their time to review manuscripts for the field, as reviewing others’ work is seen as part of scholarly citizenship – something you just do to support the field, not for personal gain. However, this can no longer be justified when publishing companies are amassing huge profits and expanding their businesses by creating new journals. Meanwhile, the same scientists who review for free then get charged for reading papers and for submitting their papers to journals.

Starting from April 2025, Computational Psychiatry (CPSY) will start testing a new model where we pay each reviewer a modest fee to compensate for their time and effort invested in reviewing manuscripts. Reviewers’ payment does not depend on the specific recommendation (accept/reject/revise) or critique they provide; instead, reviewers will be compensated per article as long as the peer review process for that article is complete. This policy is in line with most grant review processes, where a grant reviewer is compensated for their time and such compensation does not depend on the specific outcome of the grant being reviewed. This way, introducing payments will not bias the outcome of the review – a concern raised by some in the past. Along with this new policy, we will also start to publish peer review history to enhance the transparency of our peer review process. We believe these changes will not only enhance the fairness and efficiency of our peer review system but also help with the equity issue in peer review, as the lack of payment might have disproportionately affected certain types of reviewers (e.g. early career researchers, those with greater childcare burden, etc.).

A sustainable, fair, and efficient peer review system benefits affects us all and also depends on us all. We hope that this small step of starting to reimburse reviewers will lead to a better peer review system and will continue to monitor and publish journal statistics after we launch the new policy. Stay tuned with us and importantly, we hope you will contribute to the CPSY peer review process!

